# Scaffold Hunter: a comprehensive visual analytics framework for drug discovery

**DOI:** 10.1186/s13321-017-0213-3

**Published:** 2017-05-11

**Authors:** Till Schäfer, Nils Kriege, Lina Humbeck, Karsten Klein, Oliver Koch, Petra Mutzel

**Affiliations:** 1 Faculty of Chemistry and Chemical Biology, TU Dortmund University, Otto-Hahn-Str. 6, Dortmund, 44227 Germany; 2 Department of Computer Science, TU Dortmund University, Otto-Hahn-Str. 14, Dortmund, 44227 Germany; 3 Department of Computer and Information Science, University of Konstanz, Universitaetsstrasse 10, Konstanz, 78464 Germany

**Keywords:** Visualization, Chemical space, Scaffold tree, Molecule cloud, Clustering

## Abstract

The era of big data is influencing the way how rational drug discovery and the development of bioactive molecules is performed and versatile tools are needed to assist in molecular design workflows. Scaffold Hunter is a flexible visual analytics framework for the analysis of chemical compound data and combines techniques from several fields such as data mining and information visualization. The framework allows analyzing high-dimensional chemical compound data in an interactive fashion, combining intuitive visualizations with automated analysis methods including versatile clustering methods. Originally designed to analyze the scaffold tree, Scaffold Hunter is continuously revised and extended. We describe recent extensions that significantly increase the applicability for a variety of tasks.

## Background

An enormous amount of bioactivity data is already publicly available [1] and, even in academia, chemical biology and medicinal chemistry research tremendously increases bioactivity data every day. Due to advancements in technology, chemistry and biology, high-throughput screens are now applicable for a broad scientific community. Hence, the challenges in drug discovery shifted from the gathering of data to an organization and analysis issue regarding the wealth of data. Most notably, the discovery of meaningful patterns hidden in the data is a crucial and time-consuming part in recent pharmaceutical research. The underlying cheminformatics processes such as data handling, filtering or clustering can now be performed by scientists using open-source tools even without expert knowledge of the methods employed [2].

Scaffold Hunter is such a chemical data organization and analysis tool and it has been continuously enhanced since the start of its development in 2007. The platform-independent open-source tool was first released in 2009 [3] and provided an interactive visualization of the so-called *scaffold tree*, which is a hierarchical classification scheme for molecules based on their common scaffolds [4]. Since then, Scaffold Hunter was largely redesigned in order to support improved data integration and modular expandability and has progressively evolved into a flexible and comprehensive visual analytics framework for chemical compound data. The software is no longer limited to one specific analysis and visualization technique, but includes multiple interconnected views with consistent interaction mechanisms in a single user-friendly software [5, 6]. Along with this development, the scaffold tree view has been supplemented by a spreadsheet view, a plot view, and a dendrogram view based on sophisticated clustering techniques. Today, Scaffold Hunter supports a wide range of analysis tasks independent from the underlying molecular scaffolds, while scaffold based approaches still remain an integral part of the software. Scaffold Hunter allows the user to quickly switch between different representations of the same underlying data and to synchronize analysis results between these views. Therefore, Scaffold Hunter enables the user to choose the right representation for each task in the analysis process.

Most recently, three novel interactive visualizations were added to the framework. Two of these visualization techniques are based on the concept of scaffolds. The tree map view, see section “Tree map view”, provides a complementary space-filling representation to the established scaffold tree view. The molecule cloud view, see section “Molecule cloud view”, is based on the concept of Ertl and Rohde [7] and represents compound sets in a compact manner by their common scaffolds arranged in a cloud diagram. Our implementation extends the originally static concept of molecule clouds to an interactive view, which supports, among others, dynamic filtering and semantic layout techniques. Lastly, the heat map view, see section “Heat map view”, combines a matrix visualization of property values with hierarchical clustering to support the user in revealing relations between compounds and their properties.

### Related work

Several other software tools exist that address the challenges regarding the organization and analysis of chemical and biological data. Early tools such as Spotfire [8] were not originally developed to analyze these kinds of data, but are often applied to compound datasets. Simultaneously, workflow environments such as the Konstanz Information Miner (KNIME) [9], Pipeline Pilot [10] or Taverna [11] were developed. The basic idea was to enable scientists in the life sciences to perform tasks which are traditionally in the domain of data analytics specialists. KNIME additionally integrates specific cheminformatics extensions [12]. Some of them focus on the integration of chemical toolkits (e.g., RDKit [13], CDK [14], and Indigo [15]) and some others on analytical aspects (e.g., CheS-Mapper [16, 17]). CDK is likewise available in Taverna [18, 19] and Pipeline Pilot can integrate ChemAxon components [20]. Thus, these tools assist the scientists in their decision making process, e.g., which compounds should undergo further investigation. While these workflow systems facilitate data-orientated tasks such as filtering or property calculations, they lack an intuitive visualization of the chemical space. Hence, it is challenging to evaluate the results and to plan subsequent steps or to draw conclusions from a performed screen. Recently, tools tailored to the specific needs of life scientists in the chemical biology, medicinal chemistry and pharmaceutical domain were developed. These include MONA 2 [21], Screening Assistant 2 [22], DataWarrior [23], the Chemical Space Mapper (CheS-Mapper) [16, 17] and the High-Throughput Screening Exploration Environment (HiTSEE) [24, 25]. The last two tools complement the workflow environment KNIME with a visualization node. To the best of the authors’ knowledge, HiTSEE is not publicly available at present. Screening Assistant 2, CheS-Mapper and DataWarrior are open-source tools based on Java, which leads to a platform independent applicability. MONA 2 focuses on set operations and is particularly useful for the comparison of datasets and the tracking of changes. DataWarrior has a wider range of features and is beyond the scope of a pure analysis software. For example, it is capable of generating combinatorial libraries. Screening Assistant 2 was originally developed to manage screening libraries, and is able to deal with several million substances [22]. Furthermore, during import, datasets are scanned for problematic molecules or features of molecules like Pan Assay Interference Compounds (PAINS), which may disturb the assay setup or unspecifically bind to diverse proteins [26]. CheS-Mapper focuses on the assessment of quantitative structure activity relationship (QSAR) studies. Hence, it facilitates the discovery of changes in molecular structure to explain (Q)SAR models by visualizing the results (either predicted or experimentally determined). CheS-Mapper utilizes the software R [27] and the WEKA toolkit [28] to visually embed analyzed molecules in the 3D space. In summary, DataWarrior and CheS-Mapper as well as Scaffold Hunter are able to assist the discovery and analysis of SAR by utilizing different visualization techniques, see Table 1. All three tools use dimension reduction techniques and clustering methods. DataWarrior and Scaffold Hunter support a set of different visualizations in order to cope with more diverse issues and aspects regarding the raw data. Both are able to visualize bioactivity data related to chemical structures smoothly. DataWarrior utilizes self organizing maps, principal component analysis and 2D rubber band scaling to reduce data dimensionality. In contrast, Scaffold Hunter employs the scaffold concept, which provides the basis for the scaffold tree view, the innovative molecule cloud view and the heat map view, which enables the user to analyze multiple properties such as bioactivities referring to different selectivity within a protein family. Altogether, Scaffold Hunter provides an unique collection of data visualizations to solve most frequent molecular design and drug discovery tasks.

**Table 1:** Comparison of visualization techniques of cheminformatics software supporting visualization

Technique	DataWarrior	CheS-Mapper	Scaffold Hunter
Plot	Yes	–	Yes
Dim. reduction	PCA, 2D-RBS^a^, SOM	PCA, MDS	MDS
Spreadsheet	Yes	–	Yes
Clustering	Hierarchical	WEKA/R methods	Hierarchical
Special features	2D-RBS^a^	3D space, web application	Scaffold concept, collaborative features, fast heuristic clustering [29]

^a^2D rubber band scaling

## Methods

The drug discovery process requires a large amount of time, money and other resources and suffers from a small and decreasing success rate. Although automated computational methods have become standard tools to speed up this process, drug discovery essentially depends on the intuition and expertise of specialists. One important task is the analysis of chemical structures and their bioactivity data. In order to make large datasets accessible to the user, chemical structures must be organized in a meaningful way. Scaffold Hunter enables the user to explore chemical datasets following the concepts of *visual analytics*, which is the science of analytical reasoning facilitated by interactive visual interfaces [30]. In order to achieve this, Scaffold Hunter combines techniques from several fields such as data mining and information visualization to overcome an information overload caused by the mass of raw data. Visual analytics techniques aim to filter irrelevant information, present the data in a way that is easy to memorize and highlight interesting connections between entities of the data, e.g., molecules or substructures. The knowledge discovery process of the visual analytics approach is contrary to a pipeline from raw data over analysis to visualization. Instead, the goal is to allow the user to interactively explore the data, i.e., to re-adjust visualization and analysis methods in order to establish and verify hypotheses in an iterated process. To this end, Scaffold Hunter supports three core approaches, which complement each other in an analysis workflow: scaffold-based classification, clustering analysis and dimension reduction methods.

### Scaffold based approaches

The scaffold tree algorithm computes a hierarchical classification for chemical compound sets based on their common core structures referred to as *scaffolds*. Essentially, the algorithm proceeds as follows [4]: Each compound is associated with its unique scaffold that is obtained by cutting off all terminal side chains preserving double bonds directly attached to a ring. Then each scaffold is pruned by a set of deterministic rules in a stepwise fashion by removing a single ring consecutively. These deterministic rules are based on structural considerations with the aim to preserve the most characteristic core structure. The procedure terminates as soon as a scaffold consisting of a single ring is obtained. Typically, multiple molecules in a dataset share a common scaffold, and ancestors generated in the successive process of simplification coincide. The scaffold tree is constructed by merging the scaffolds that occur more than once. Scaffolds that are not directly obtained from any molecule of the collection, but generated by the pruning process, are said to be *virtual*. Virtual scaffolds provide promising starting points for the synthesis or acquisition of compounds complementing the current collection [3]. For a detailed description of the underlying algorithm the reader is referred to Schuffenhauer et al. [4].

The concept of scaffolds is fundamental for Scaffold Hunter and forms the basis for several visualizations. This includes the scaffold tree view, see section “Established views”, which was the central view of the first release of Scaffold Hunter in 2009 [3]. Recently, two novel visualization techniques based on scaffolds were implemented: the tree map view and the molecule cloud view, see sections “Tree map view” and “Molecule cloud view”, respectively.

### Clustering techniques

Clustering is a well-established unsupervised learning technique that serves as an alternative classification scheme to the scaffold based approach. Rather than identifying most characteristic substructures, clustering methods analyze the similarity structure of a dataset and group similar molecules in so-called *clusters* while assigning dissimilar molecules to different clusters. So, clustering methods support the user in various use cases, such as getting an overview over large datasets or analyzing relations between different molecular properties. For example, a clustering based on structural features which aligns with an annotated property indicates the presence of a connection between the used structural features and the property. The unsupervised nature of the clustering method allows the applicability to use cases, where no pre-defined classification scheme exists or the existing classification schemes do not fit the task at hand. Scaffold Hunter provides various similarity measures, that are based on the molecular structure, chemical *fingerprints*, i.e., abstract bitstring representations of a molecule’s structural characteristic, or annotated properties and enables the user to cluster a dataset according to different aspects. The resulting hierarchy is often visualized as a so-called *dendrogram*, see Fig. 6. Although a wealth of different clustering algorithms exists, Scaffold Hunter focuses on sequential agglomerative hierarchical non-overlapping (SAHN) clustering [31]. In contrast to flat clustering methods, which output a partitioning of the dataset, SAHN methods produce a similarity hierarchy, i.e., they present various flat clusterings with different granularities at once. SAHN methods enable Scaffold Hunter to adjust the clustering granularity interactively without the need to specify a granularity level beforehand. While SAHN methods provide a deep insight into the similarity structure of a dataset, they are computationally demanding for larger datasets. To overcome this burden, Scaffold Hunter provides a fast heuristic SAHN method [29], which is easily configurable, intuitive to use and applicable to several hundreds of thousands of molecules.

The dendrogram view, see section “Established views”, and the novel heat map view, see section “Heat map view”, are both based on SAHN clustering methods.

### High-dimensional data visualization and low-dimensional embeddings

Chemical data is typically high-dimensional, e.g., a large number of numerical properties can be associated with each molecule. Moreover, chemical fingerprints encode the presence or absence of a very large number of structural features [32]. Therefore, a direct visual inspection of data in such spaces is often not feasible and does not provide any deeper insight into the similarity structure of the data. A straight-forward way to reduce the dimensionality is to manually select a few dimensions (or properties) of interest. For example, a 2D plot maps two molecular properties to the plot’s axes and displays the molecules as dots on the plane. The visualization may intuitively reveal relative dissimilarities, clusters of similar molecules and correlations between the properties. The number of displayable dimensions can be increased with the help of different colors, shapes, sizes and rotatable projections. Scaffold Hunter makes extensive use of these possibilities as described in section “Realization”. However, adequate visualization and perception is still limited to a very low-dimensional space [33].

In addition to the limited number of dimensions that can be visualized at the same time, not all types of data can be directly represented as finite numerical vectors. For example, there are molecular similarity measures directly defined on the molecular graph structure. In these cases, pairwise similarities are often the only possibility to represent the similarity structure of a dataset. In both situations, i.e., high-dimensional or non-vectorial data, a projection onto a lower dimensional space is desirable for visualization. In general, an isometric embedding, i.e., an embedding that preserves the distances, is not possible. Thus, the major goal in our use case is to preserve the relative distances, such that similar data points are embedded close to each other and dissimilar data points are placed far apart. There are several well-established methods for this task, such as principal component analysis (PCA), self organizing maps (SOM), multi dimensional scaling (MDS) or generative topographic mappings (GTM) [34, 35]. In the context of semantic word clouds several additional methods—such as Seam Carving, Inflate & Push, Star Forest or Cycle Cover [36] have emerged, which try to realize a compact representation as a secondary criterion. The novel molecule cloud view described in section “Molecule cloud view” is based on these concepts.

## Realization

No single data visualization will satisfy all user requirements independent of the type of data and tasks at hand. Scaffold Hunter thus provides several visualizations, including established techniques from information visualization, as well as visualizations tailored towards the specific requirements of compound analysis. Three novel views are the most preeminent recent innovations of Scaffold Hunter: (i) the tree map view, see section “Tree map view”, (ii) the heat map view, see section “Heat map view” and (iii) the molecule cloud view, see section “Molecule cloud view”. Before we describe them in detail, we will give a short summary of Scaffold Hunter’s architecture in section “Architecture” and of the established views in section “Established views”.

### Architecture

**Fig. 1:**
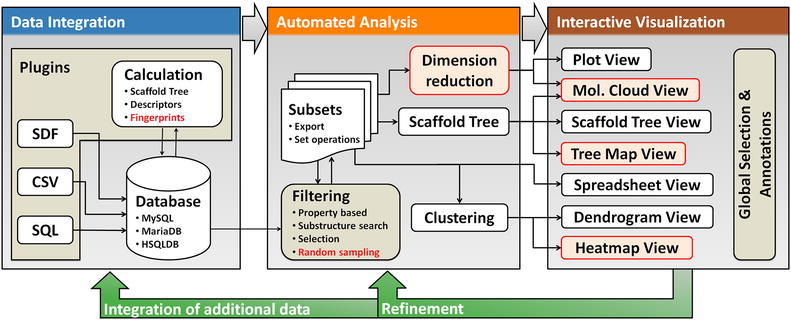
The architecture of Scaffold Hunter supports the knowledge discovery process from data integration to data analysis up to interactive visualization of the results. The diagram extends a figure previously published in [6], recent innovations are highlighted in *red*

Scaffold Hunter’s modular architecture comprises three main building blocks that support data integration, automated analysis, and interactive visualization for use in a cyclic knowledge discovery process, see Fig. 1. Support for different data import formats, data transformations, and property calculations is realized using a plugin concept, and thus can easily be extended to new formats and properties. The current version of Scaffold Hunter already supports several built-in property calculators, most notably for structural fingerprints. Datasets can be merged and extended by additional data at a later stage, e.g., when new experimental or public information is available. After import the data can be processed and analyzed using filtering, clustering, dimensionality reduction and by applying the scaffold tree approach. The results can then be visualized in any of the interactive views provided by the framework for visual inspection and further selection.

The visualizations can be used in combination, which allows the user to profit from their complementary strengths. Linking via a global selection and filtering mechanism facilitates to switch between visualizations, and a flexible subset storage mechanism allows the user to organize the compound set and to either compare different datasets using a single visualization or to analyze different aspects of a single dataset with the help of different types of views. All views support a mapping of compound or scaffold properties to visual cues, e.g., color, and the visualizations can be annotated with comments to support collaboration or persistently store findings generated during the workflow. Detailed information on a scaffold or compound is displayed in tooltip windows that pop up when hovering the mouse over the corresponding element.

### Established views

We briefly summarize the established visualizations provided by Scaffold Hunter and refer the reader to our previous publications [3, 5, 6] for further details.

#### Scaffold tree view

**Fig. 2:**
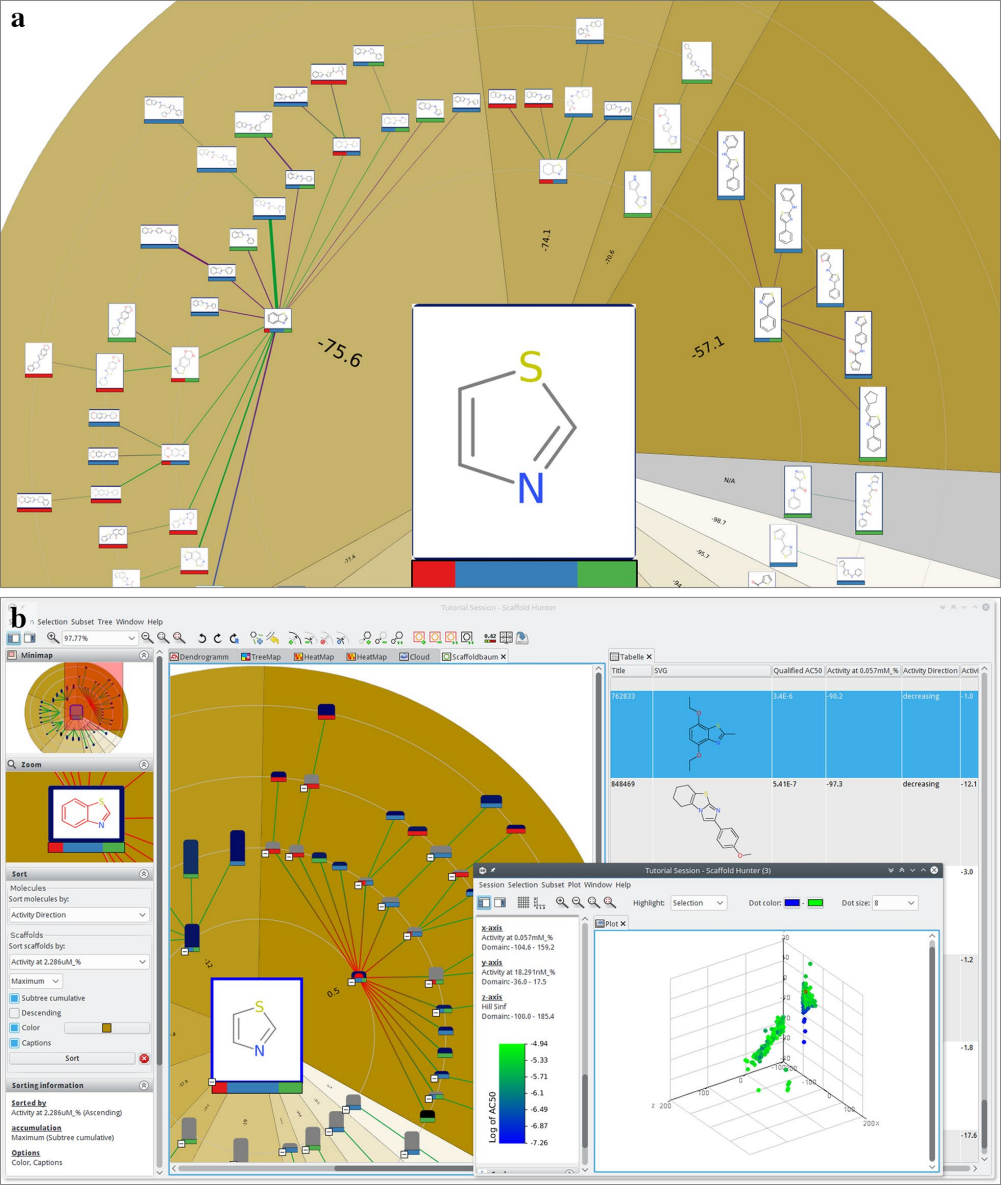
Close-up of a branch of the scaffold tree (**a**) and multiple linked views (tree, spreadsheet and plot view) showing the same dataset simultaneously (**b**)

The scaffold tree view allows interactive exploration of the scaffold tree structure described in section “Scaffold based approaches”. Using a classical radial tree layout for the visualization, this view allows the user to gain an overview on the structure classification hierarchy as well as the distribution of structures within the dataset, and serves as a starting point for the search workflow. Figure 2a shows the scaffold tree view with a user-defined sorting of the scaffolds based on some scaffold property, as well as a mapping of further properties to the node background color.

#### Dendrogram view

The hierarchy resulting from the clustering techniques described in section “Clustering techniques” can be represented by a binary tree and is often visualized as a dendrogram, where the height of each inner node in the tree corresponds to the similarity of the two child clusters, see Fig. 6 for an example. Scaffold Hunter provides a dedicated dendrogram view that realizes an enhanced version of the standard dendrogram visualization, adapted to the requirements of compound analysis. It features a combined dendrogram and spreadsheet configuration that allows a detailed analysis of the clustered molecules within a limited screen estate. The user can interact with the dendrogram view to navigate in the hierarchy and by panning and zooming, and to select a level in the cluster hierarchy for highlighting.

#### Molecular spreadsheet and plot view

Scaffold Hunter also provides a spreadsheet and a plot view to visualize information on the molecules in the database. The spreadsheet view provides detailed molecule information shown in a tabular visualization including molecule properties, as well as their names, SVG-images and user-defined flags. It implements the standard functionalities such as sorting by a user-selected column, reordering of columns, pan and zoom with the minimap, sticky columns, and cell resizing. Annotations such as comments and flags can be changed via the spreadsheet view. The plot view allows the user to visualize relations between molecule properties in 2- and 3-dimensional scatter plots. Beyond the mapping of properties to coordinates, additional properties can be mapped to color or size to allow the user to visualize more than three properties at a time, see Fig. 2b for an example of a color mapping.

### Tree map view

**Fig. 3:**
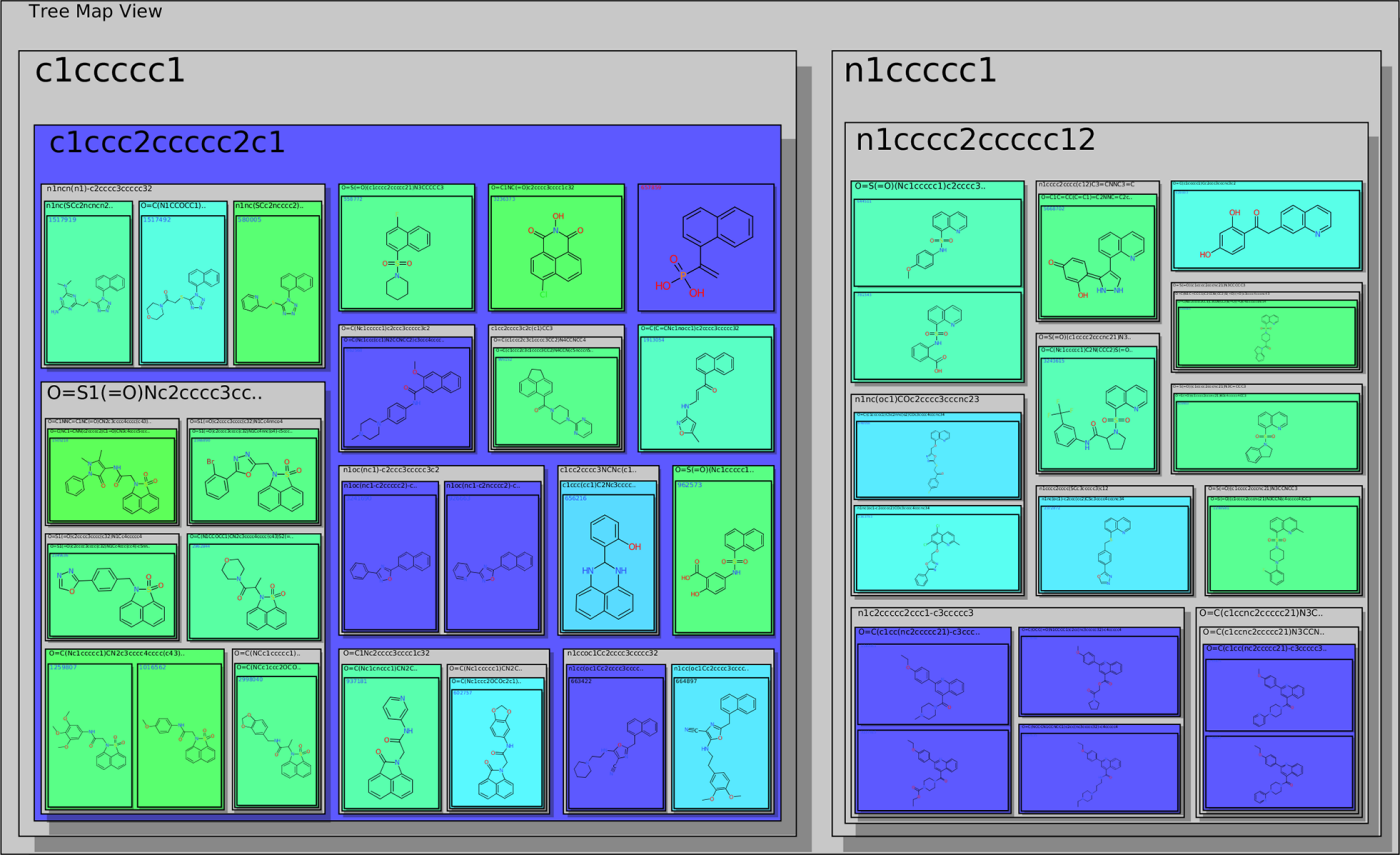
The tree map view with property values mapped to *background colors*. Shown are parent scaffolds, the *nested rectangles* represent child scaffolds

The tree map view visualizes the scaffold tree described in section “Scaffold based approaches” in a space-filling manner. It provides an alternative to the scaffold tree view and expands the applicability of Scaffold Hunter to use cases for which this view turned out to be less suitable. In contrast to the scaffold tree view, the tree map view does not employ a classical tree layout, where the relation between nodes is represented by edges. Instead, the scaffold tree structure is illustrated by nested rectangles, and each scaffold in the tree is represented by a rectangle. The molecules associated with the scaffold are drawn within this rectangle as structural formulas and the children in the scaffold tree are represented by nested rectangles, see Fig. 3. This space-filling approach to visualize trees is referred to as *tree map* and was first proposed in [37]. Compared to the radial layout of the scaffold tree in Fig. 2a, this approach provides a compact representation making optimal use of the available screen space. Moreover, while the classical scaffold tree view focuses on the scaffolds itself, the tree map view uses the scaffold tree primarily as a hierarchical classification scheme to group molecules by their common scaffolds. As a consequence, the size of an encompassing rectangle directly corresponds to the number of molecules it contains and, thus gives an intuitive impression of the fraction of molecules in the dataset that are related to the associated scaffold. As an alternative, it is possible to scale the depiction of molecules relative to their property values. The size of the encompassing rectangles is adjusted accordingly and the screen space occupied by a subtree reflects its relative importance with respect to the property. For very large datasets, the tree map view optionally displays the leafs of the scaffold tree only instead of the molecules, which reduces the size of representation.

Our visualization uses clearly visible shaded frames with title lines and padding to the neighboring rectangles to highlight the nested structure. Furthermore, this makes the background of all rectangles visible, which is used to encode property values by colors just as for the scaffold tree view. When displaying the molecules of a dataset and mapping a property of the molecules to the background color, the encompassing rectangles can be colored according to the average, minimum or maximum value of either all the contained molecules or just those directly associated with the corresponding scaffold. This gives an easily comprehensible impression of the heterogeneity with respect to the selected property at a glance and indicates how well it aligns with the scaffold based grouping. The tree map view allows zooming and supports to pan by grab and drag consistent with all other views. Furthermore, it is possible to focus an encompassing rectangle by clicking on its title. This triggers an animated zooming operation and supports the systematic explorations along branches of the scaffold tree.

### Molecule cloud view

**Fig. 4:**
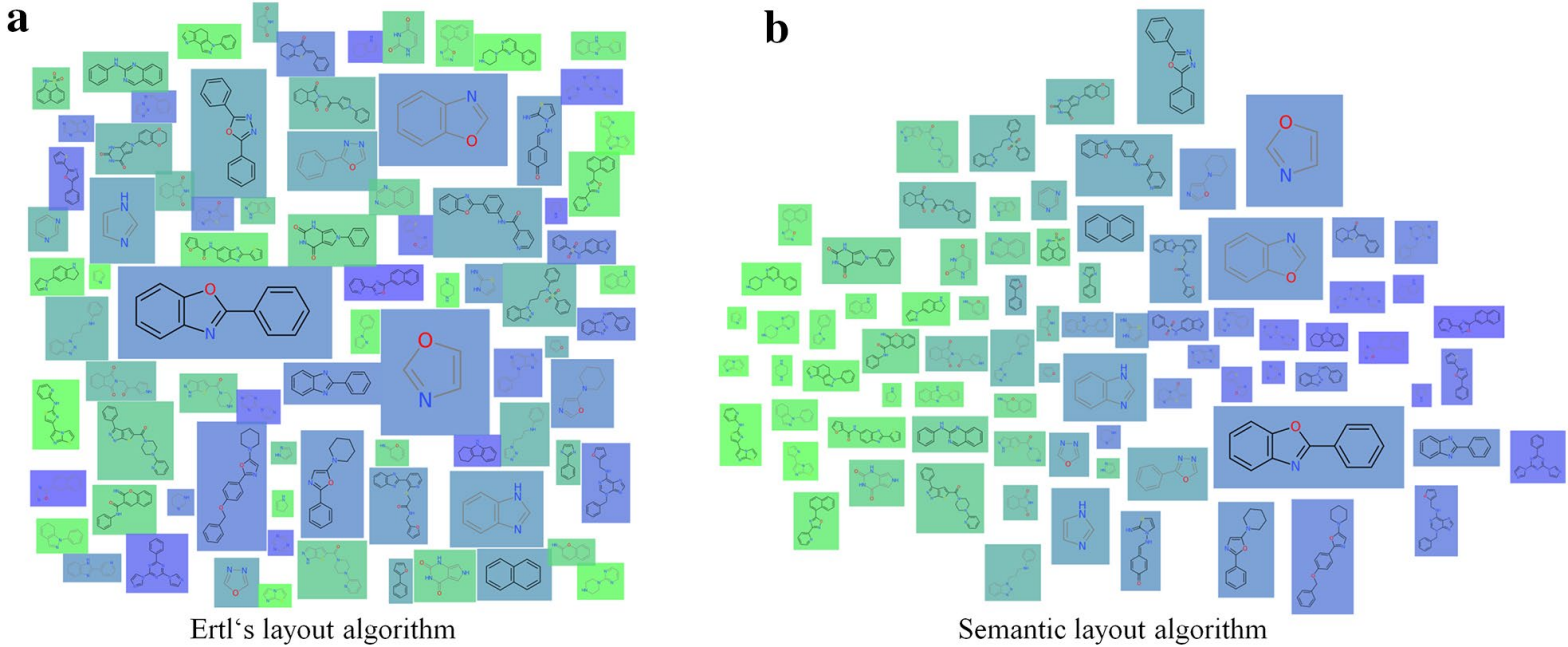
A molecule cloud consisting of 81 scaffolds, colored according to qualified $\hbox {AC}_{50}$ values (*blue*—low, *green*—high). The layout (**a**) places large scaffolds in the center of the cloud, the semantic layout (**b**) is configured to place scaffolds with similar property value close to each other

The concept of a *molecule cloud* is inspired by the widely used *word clouds* and has been introduced to cheminformatics by Ertl and Rohde [7] to provide compact diagrams summarizing a collection of molecules. The approach relies on the concept of scaffolds described in section “Scaffold based approaches”: the elements constituting the molecule cloud are the scaffolds obtained from a set of molecules, whereas each scaffold is scaled according to the number of molecules it represents. These scaffolds are drawn compactly on the plane to form a cloud diagram. To this end, Ertl et al. [7] proposed a layout algorithm, which first places large scaffolds at the center and then aims at arranging the remaining scaffolds, such that the gaps are filled, see Fig. 4a. Our realization in Scaffold Hunter extends the original concept by supporting user interaction and semantic layout algorithms, which take molecular similarities into account.

#### Interactive realization

 In Scaffold Hunter an interactive view was developed, which uses the static concept of molecule clouds as a basis. Just like several other existing views the cloud view allows to zoom and pan, which is particularly essential for large datasets. Properties of molecules and scaffolds can be represented by the background color, see Fig. 4. For this purpose, properties of scaffolds can be computed as the average, minimum or maximum value of all molecules associated with them. The cloud view benefits from the subset concept described in section “Architecture” like any other view. In addition, interactive refinement of the molecule cloud is supported by filtering out scaffolds, e.g., according to the number of molecules associated with them or their number of rings. The filter criteria can quickly be adjusted by sliders on the left side bar, which control the minimum and maximum values. The view can be configured to automatically recompute the layout after each filtering step in order to keep the representation as compact as possible and close the emerging unused space. To achieve this efficiently, a new dynamic layout algorithm was developed, which avoids the re-computation from scratch and at the same time aims at preserving the user’s mental map. This is achieved by minimizing the change between two different layouts in combination with smooth animated layout transitions.

#### Semantic layouts

**Fig. 5:**
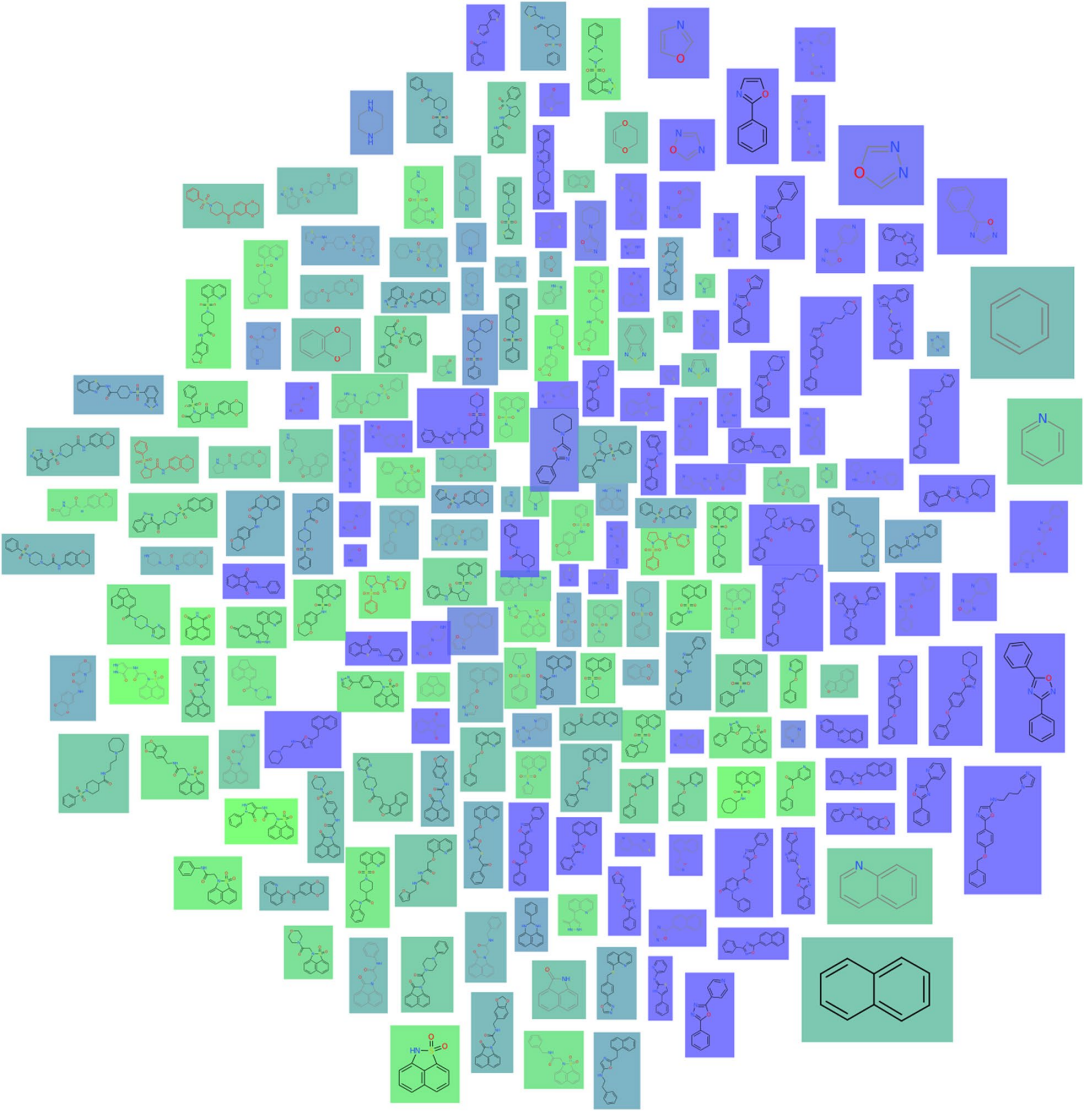
Molecule cloud view with semantic layout: similarities are obtained from the Tanimoto coefficient between ECFP4 fingerprints, the background color represents the property “Hill Sinf” averaged over the associated molecules (*blue*—low, *green*—high)

 Scaffold Hunter supports different layout algorithms for the cloud diagram and the user can switch between them at any time. First, the original layout algorithm kindly provided by Ertl [7] was integrated. Moreover, layout algorithms from [36] were incorporated, which were originally proposed for so-called *semantic word clouds*: The key idea of these approaches is to place semantically related words close to each other. In the domain of molecules our goal is to place similar scaffolds close to each other, whereas the user can choose from various similarity measures. These are derived from a specific property (or a set of properties) or obtained by standard cheminformatics techniques such as the Tanimoto coefficient applied to structural fingerprints. The user can quickly switch between different similarity measures giving rise to animated changes of the layout. The effect of the semantic layout algorithms is illustrated in Fig. 4b, where the same property used to define the background color is also used as basis for the similarity calculation. The scaffolds are arranged such that green scaffolds with a high property value are placed on the left and those with a low value on the right giving the impression of a color gradient, which indicates that the layout preserves the similarities well. However, similarities and distances stemming from high-dimensional data cannot be embedded in the plane without severe distortion, see section “High-dimensional data visualization and low-dimensional embeddings”. The primary objective was to provide a compact diagram without gaps and to consider similarities only as a secondary criteria. In this respect, our approach differs from many techniques widely-used in cheminformatics [34] to map high-dimensional molecular data into a low-dimensional space for visualization. Nevertheless, this technique can be particularly useful when the similarity is defined based on structural properties, e.g., obtained from fingerprints, and the background color represents a numerical property of interest, e.g., the biological activity: in this case, the molecule cloud can give hints on the relation between structure and activity. Figure 5 shows a cloud diagram obtained for this setting. The region on the left-hand side is predominantly populated by green scaffolds (high property values), while blue scaffolds (low property values) are placed on the right half of the diagram. However, there are also individual blue scaffolds on the left, which may indicate the presence of so-called *activity cliffs* [38], i.e., structurally similar molecules with a significant difference in potency. In contrast to the results obtained by clustering, the visualization does not necessarily partition elements into distinct groups, but represents pairwise similarities in an intuitively comprehensible manner.

### Heat map view

Scaffold Hunter provides a heat map view to show relations between compounds and their properties. Some computer science aspects of the approach have previously been discussed in [39]. It is best suited to discover (partial) correlations between compound similarity and different properties as well as outliers with respect to compound similarity. A heat map is a visual representation of a 2-dimensional matrix. It is composed of rectangular tiles, colored or shaded, where each tile represents a matrix entry and is positioned in accordance to the matrix indexes. The color of each tile is defined by a mapping of the value of the associated matrix entry. This way of coloring matrices has its roots in the 19th century [40] and gives a quick graphical overview over the matrix value distribution. The matrix displayed in the heat map view contains property values. Each column of the matrix is associated with a compound and each row with a property.

**Fig. 6:**
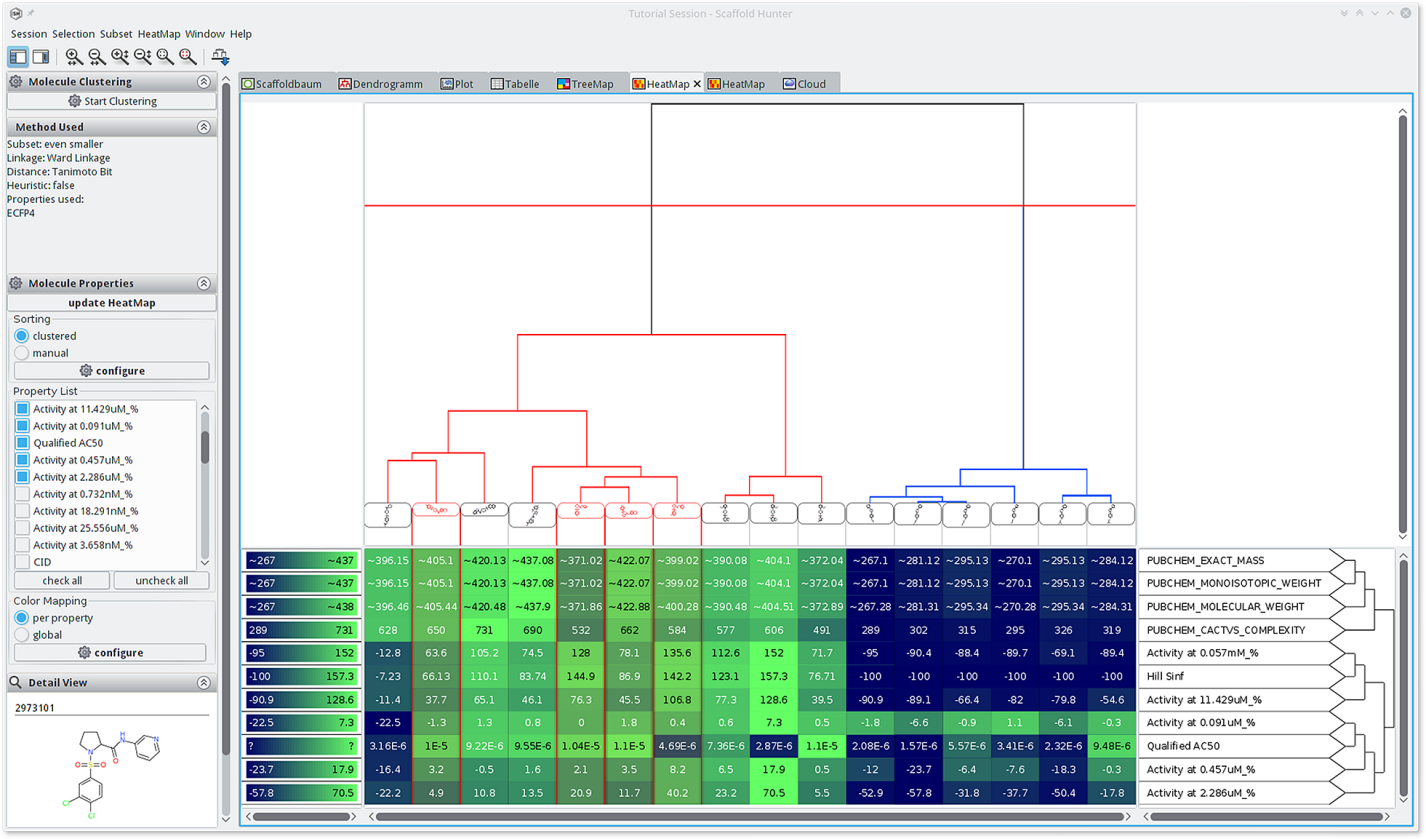
Heat map view showing a clustering based on ECFP4 fingerprints revealing correlations with several activity scores. On the *top* of the heat map it shows a dendrogram, which is the result of the clustering based on structural ECFP4 fingerprints. A two dimensional depiction of the molecular structure is attached to each leaf node of the dendrogram. The *red horizontal line* shows a similarity threshold that separates the dataset into a *red* and *blue* cluster of compounds. On the *right hand side* of the heat map the property names and a dendrogram over their similarity is displayed. A legend for the heat map color mapping is presented on the *left hand side* of the heat map

In order to identify correlations between the compound similarity and the property values, it is important that the matrix is ordered in a way such that similar compounds are close to each other. In this case, correlations can be identified by consecutive tiles with similar colors. Analogously, an appropriate ordering of the properties enables the user to discover correlations between different properties. For this reason, a heat map is often ordered with the help of a dendrogram (see section “Clustering techniques”) stemming from a hierarchical clustering algorithm. It is important to notice that a dendrogram induces multiple linear orderings that are consistent with its binary tree structure. Neighbors in such an ordering do not need to be similar, especially if they stem from different high level clusters. Depicting the dendrogram beside a heat map therefore reveals important additional information with respect to the similarity structure of the dataset. Figure 6 shows an example of such a situation inside the heat map view of Scaffold Hunter. In order to calculate the similarity of two properties, the heat map rows are interpreted as numerical vectors in the Euclidean space and are clustered using a single link SAHN algorithm [29]. Missing values get replaced by their expected value with respect to other values of the same property. Thus, it is possible to display a heat map on partially defined properties. The heat map shown in Fig. 6 quickly reveals the correlation of several attributes with respect to the clustering structure. This is indicated by the different average colors for the heat map cells associated with the red and the blue cluster. It also reveals an outlier of the property *Qualified* $\mathrm{AC}_{50}$ (3rd row from below) for the second from the right compound of the red cluster, i.e., the $\hbox {AC}_{50}$ value is unexpectedly low for the red cluster.

**Fig. 7:**
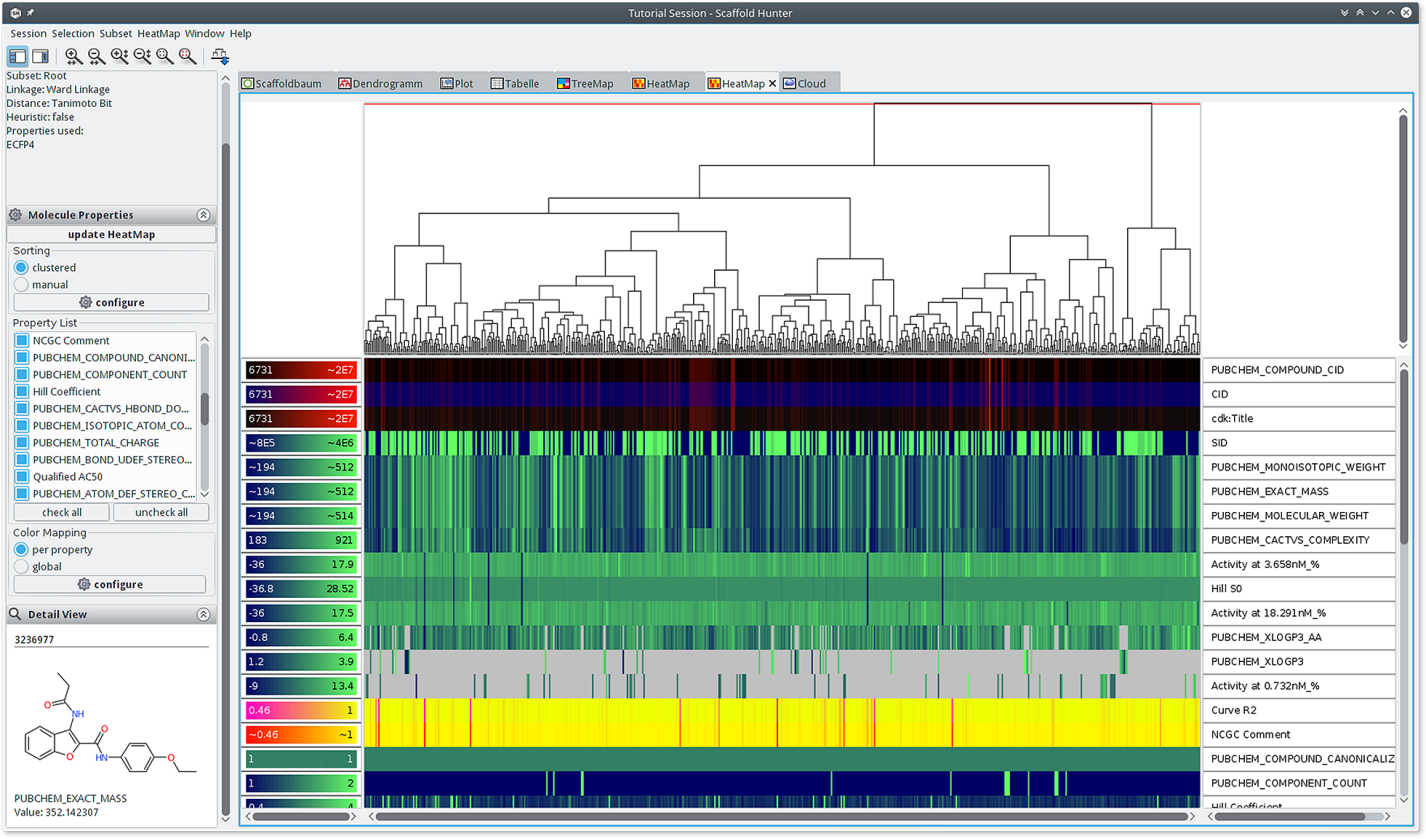
Heat map view with individual color mapping and reduced level of detail as a result of the semantic zoom

Scaffold Hunter’s heat map view is highly configurable and allows the adjustment of the matrix ordering, the visual appearance, and the level of detail to individual needs. The hierarchical clustering configuration allows the selection of various similarity measures, linkages, fingerprints and numerical properties. As an alternative to the matrix ordering induced by a clustering, it is possible to adjust the ordering of the properties manually using drag and drop. Utilizing the left sidebar one is able to filter out irrelevant properties. To support the visualization of larger datasets, the heat map view supports a semantic zoom, which automatically adjusts the level of detail displayed by a single tile. When a tile is large enough it displays the exact value of the property. For lower zoom levels the properties are rounded or hidden and the cells are rendered without borders as shown in Fig. 7. Still, the exact value and molecular depiction can be quickly accessed in the details section of the left sidebar by hovering a tile with the mouse. In order to handle different property types and scales, Scaffold Hunter provides a manual adjustment of the value to color mapping for each individual property. Scaffold Hunter offers gradient or interval based color mappings with adjustable ranges. This feature is also highlighted in Fig. 7, where different color palettes show different color mapping configurations.

### Other recent developments

Besides the integration of novel views described above, Scaffold Hunter has been extended by various new features and improved in many ways. In the following, the most important changes are summarized. Several performance improvements have affected multiple modules of Scaffold Hunter. This includes several visualizations, the dataset import and export as well as the calculation of fingerprints and properties. The handling of SD files—especially if they do not fully comply with the technical standard—has become much more robust. Scaffold Hunter now supports the calculation of additional properties for molecules as well as for scaffolds. This has been achieved by a plugin mechanism to support easy extension, which is now also applicable to scaffolds. Among others we have incorporated a plugin for the calculation of the popular extended connectivity fingerprints (ECFP/FCFP) [41] in various configurations. Recent versions of Scaffold Hunter support chirality and it is possible to individually select whether chiral information is taken into account, e.g., for molecular matching and depiction. The molecular depiction now also offers a cleaner visual appearance with more colors and a better layout of complex structures. Finally, the subset management has been improved and additional operations to generate subsets are now available. Scaffold Hunter supports random sampling of subsets and an operation to split a subset according to its scaffold tree structure into smaller parts.

## Results and discussion

### Case studies using the established visualization techniques

Scaffold Hunter supports the user in analyzing chemical spaces, for example, those of natural products [42] and large chemical datasets. It has already proven to be useful for various research tasks such as scaffold hopping, target prediction, chemical space analysis and natural product simplification. Natural products are considered as biologically relevant classes with a limited synthetical accessibility. By brachiating along the branches of a natural product scaffold tree, simpler molecules can be identified with similar bioactivity but higher synthetical accessibility [43]. This brachiation is particularly useful to reduce complex structures to receive natural product inspired compounds which are synthesizeable and retain bioactivity [44]. The virtual scaffolds are particularly good starting points for synthesis if the parent and child scaffolds exhibit similar bioactivity. In one of the first applications of Scaffold Hunter, these virtual scaffolds were utilized to discover novel pyruvate kinase modulators [3]. Four virtual scaffolds, identified in a pyruvate kinase assay dataset, served as the basis for a dataset containing compounds possessing one of these virtual scaffolds as substructure. A hit rate of 10% in subsequent tests for pyruvate kinase modulatory function proved this approach as successful. In another analysis, this approach led to a 5-lipoxygenase inhibitor with a novel scaffold and higher ligand-efficacy than the corresponding child scaffold and novel estradiol-like estrogen receptor modulators [42]. In addition, by merging non-annotated natural products with annotated synthetic molecules Scaffold Hunter can assist to assign a potential target protein to natural products or their simpler scaffolds along a branch. By merging the $\gamma$-pyrone branch of the Dictionary of Natural Products [45] and the Wombat database [46]. Wetzel et al. [47] were able to identify new inhibitors for several proteins. This approach resulted in a novel class of nonpeptidic small molecule STAT inhibitors which was independently discovered by a high-volume screening approach by Berg et al. [48]. For acid sphingomyelinase (aSMase) a novel scaffold which exhibits better properties like a lower AlogP value could be identified which do not inhibit the neutral isoform. A third class of proteins which can be targeted by $\gamma$-pyrones are monoamine oxidases (MAO). In this case Scaffold Hunter was able to unveil subtype preferences of the corresponding scaffolds. In a similar analysis, a potential target in the antiparasitics domain could be proposed by merging the kinase SARfari data [49] provided by ChEMBL [50] with screening data against trypanosomatid parasites [6].

### Case studies using the novel visualization techniques

**Fig. 8:**
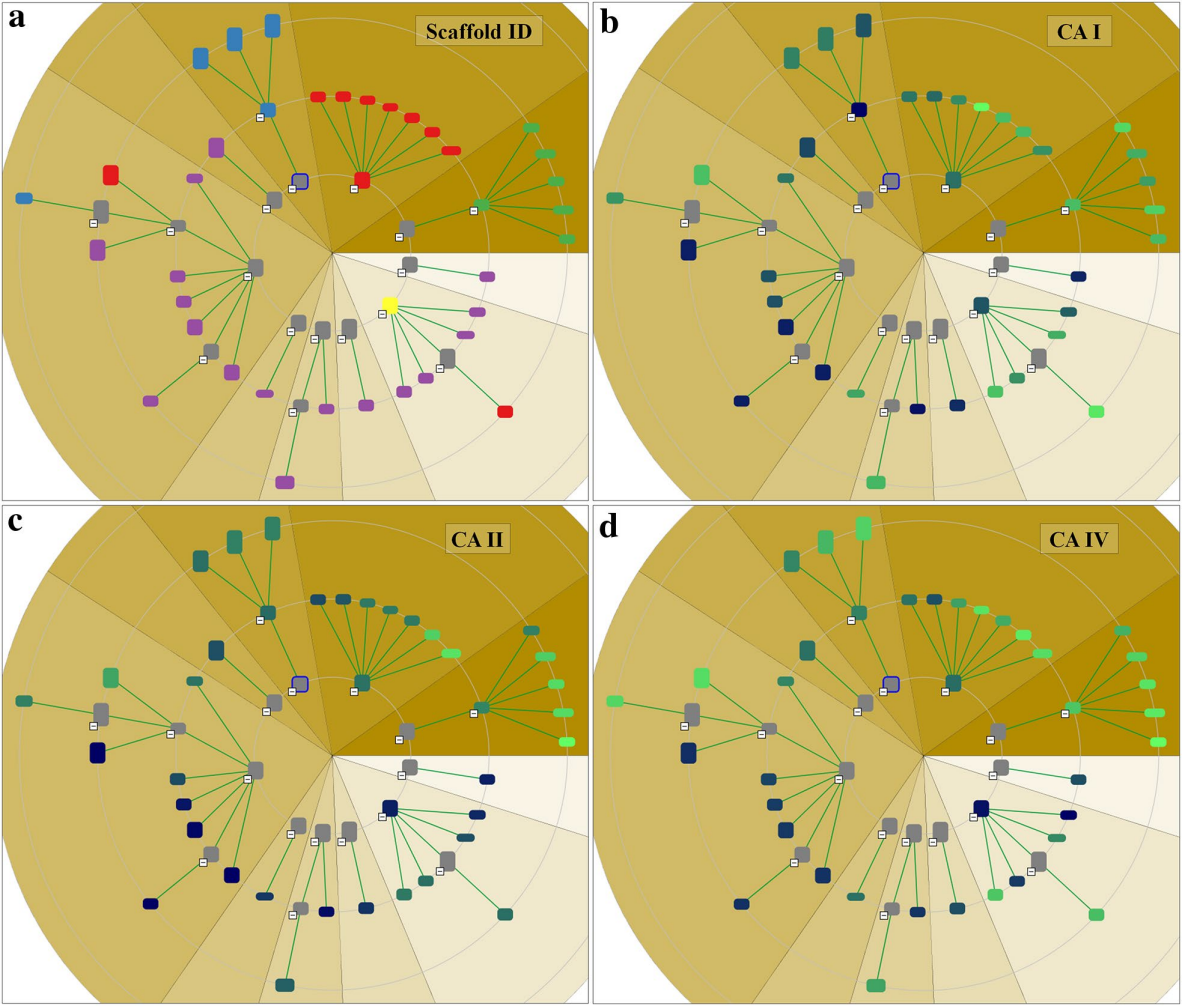
The scaffold tree view of a dataset of carbonic anhydrase inhibitors. *Colors* indicate the scaffold id (**a**) and the strength of activity against CA I (**b**), CA II (**c**) and CA IV (**d**)

For a case study to exemplify the use of the novel implemented views, a dataset of 87 carbonic anhydrase (CA) inhibitors was analyzed with Scaffold Hunter. This dataset was assembled by Weber et al. [51] to create 3D QSAR models that could explain the determined selectivities between three CA isoforms (CA I, CA II and CA IV). The structures including activity data are available for download under doi:10.17877/DE290R-17935. The dataset contains molecules from 6 distinct chemical scaffolds (synthetic routes) that were tested on two cytosolic forms: CA I, CA II and one membrane-bound form: CA IV. The authors stated especially that: (i) scaffold 6 is higher active on the CA II and CA IV than on CA I (ii) the descending order of correlation is: CA II and CA IV > CA I and CA IV > CA I and CA II (iii) the descending order of strength of activity is: CA II > CA IV > CA I (iv) they predicted compounds A13 and D16 too good for CA I and CA II in some cases for D16. These results could be reproduced with Scaffold Hunter. First, the scaffolds generated by Scaffold Hunter mostly correspond to the scaffold assignments from Weber et al. which is demonstrated by the scaffold tree view. Therefore, the nodes of the scaffold tree were colored according to the scaffold id assigned by Weber et al., see Fig. 8a. Additionally, the scaffold tree was sorted according to the pKi values of CA II to get a first impression of the activity distribution through the scaffolds. Scaffold 1 (red), scaffold 2 (blue) (with few exceptions) and scaffold 3 (green) are grouped together in the scaffold tree. Scaffold 5 (orange) and 6 (yellow) are pooled together with scaffold 4 (purple) in the benzene scaffold. This is explicable due to the absence of more discriminative ring systems than a benzene. In fact, scaffolds 5 and 6 do not contain any ring system. However, the scaffold classification of Scaffold Hunter is reasonable as it correlates with the inhibitory activities, see Fig. 8b–d.

**Fig. 9:**
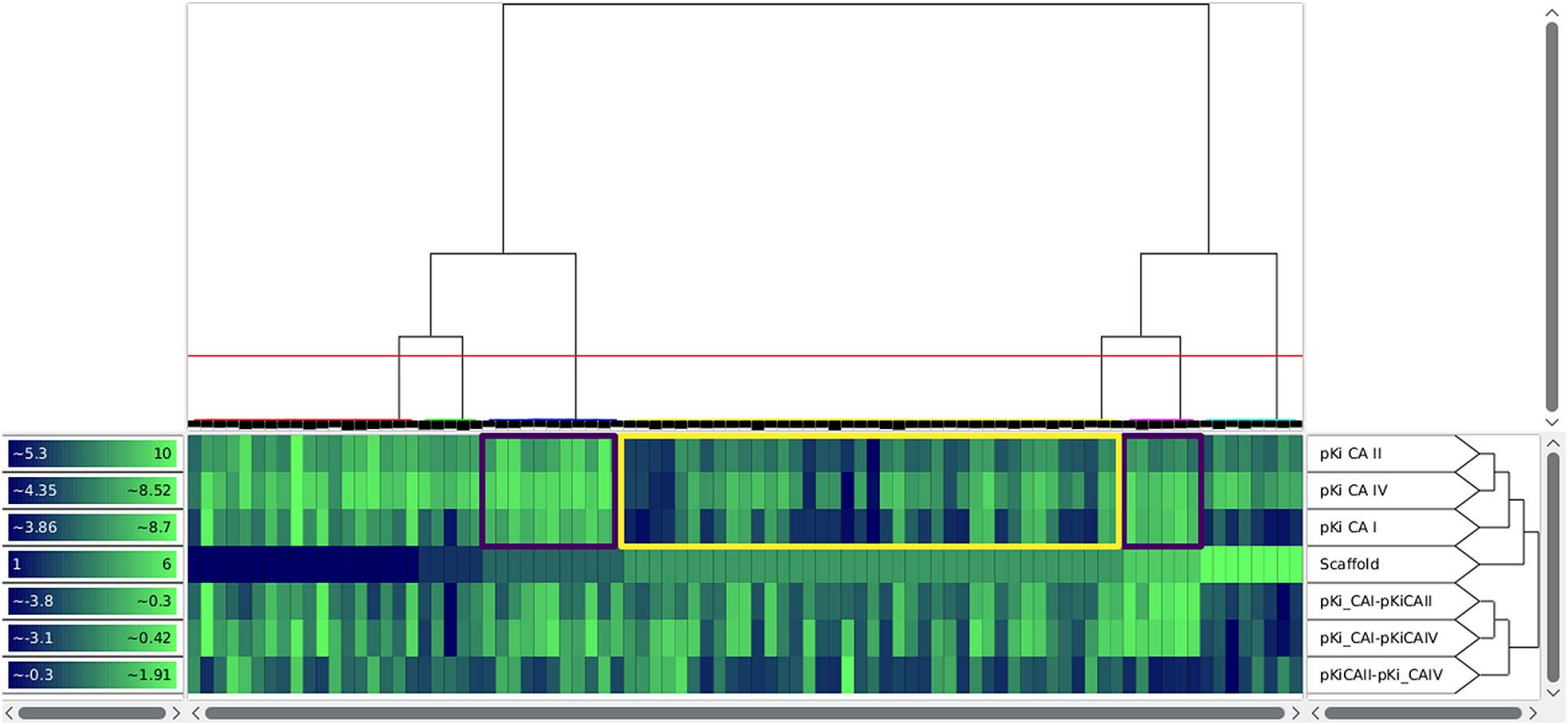
The heat map view which is clustered according to the scaffold id originating from Weber et al. support the proposed assessment of the scaffolds. *Colored boxes* are not created by Scaffold Hunter

To prove the statements, the molecules were clustered according to their scaffold id from the original publication and the activities were visualized as a heat map, see Fig. 9. Scaffold 3 and 5 (purple boxes) are more active than the others, and scaffold 4 (yellow box) is less active, which is in accordance to Weber *et al*. One clearly recognizes that scaffold 6 (right part next to right purple box) is less active on CA I. This view furthermore supports that the activities for CA II and CA IV are most related because they are earlier condensed in the dendrogram on the right. Additionally, the order of activity strength though the isoforms can be estimated visually by evaluating the color components, thereby one has to take into account the different ranges of the color gradient on the left side of each row.

**Fig. 10:**
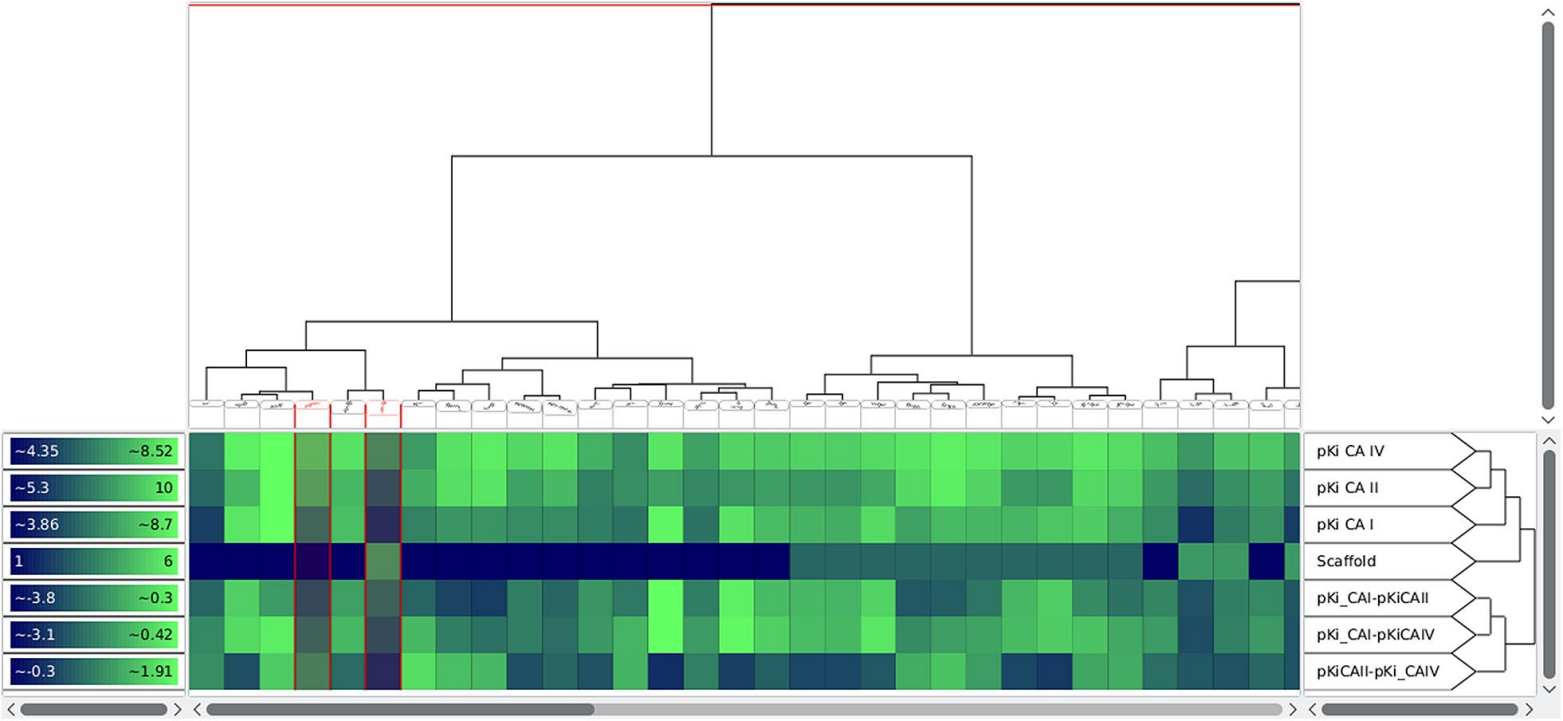
The heat map view which is clustered according to the ECFP4 reveals activity differences though structural similarity

The deviations of predicted and measured pKi values for A13 and D16 can be explained by activity cliffs or a weaker form, i.e., structurally similar compounds exhibit activities which differ more than expected. In Scaffold Hunter structural fingerprints such as the ECFP4 can be calculated and subsequently used to cluster the compounds w.r.t. their structural similarity to reveal activity cliffs, see Fig. 10. Subsequently, the neighbors of A13 and D16 (selected in Scaffold Hunter and thus red-rimmed) were analyzed. For A13 (left marked column) structurally similar compounds were A10 as well as A11 (left to A13), for D16 (right marked column) A12 (left to D16) is structurally similar which is in good accordance with the results from Weber et al. Nevertheless, the structurally similar molecules to A13 and D16 are more active (more visibly green), which leads to the overprediction observed by [51]. In summary, the heat map indicates that, despite the structural similarity, the activities differ, see Fig. 10.

**Fig. 11:**
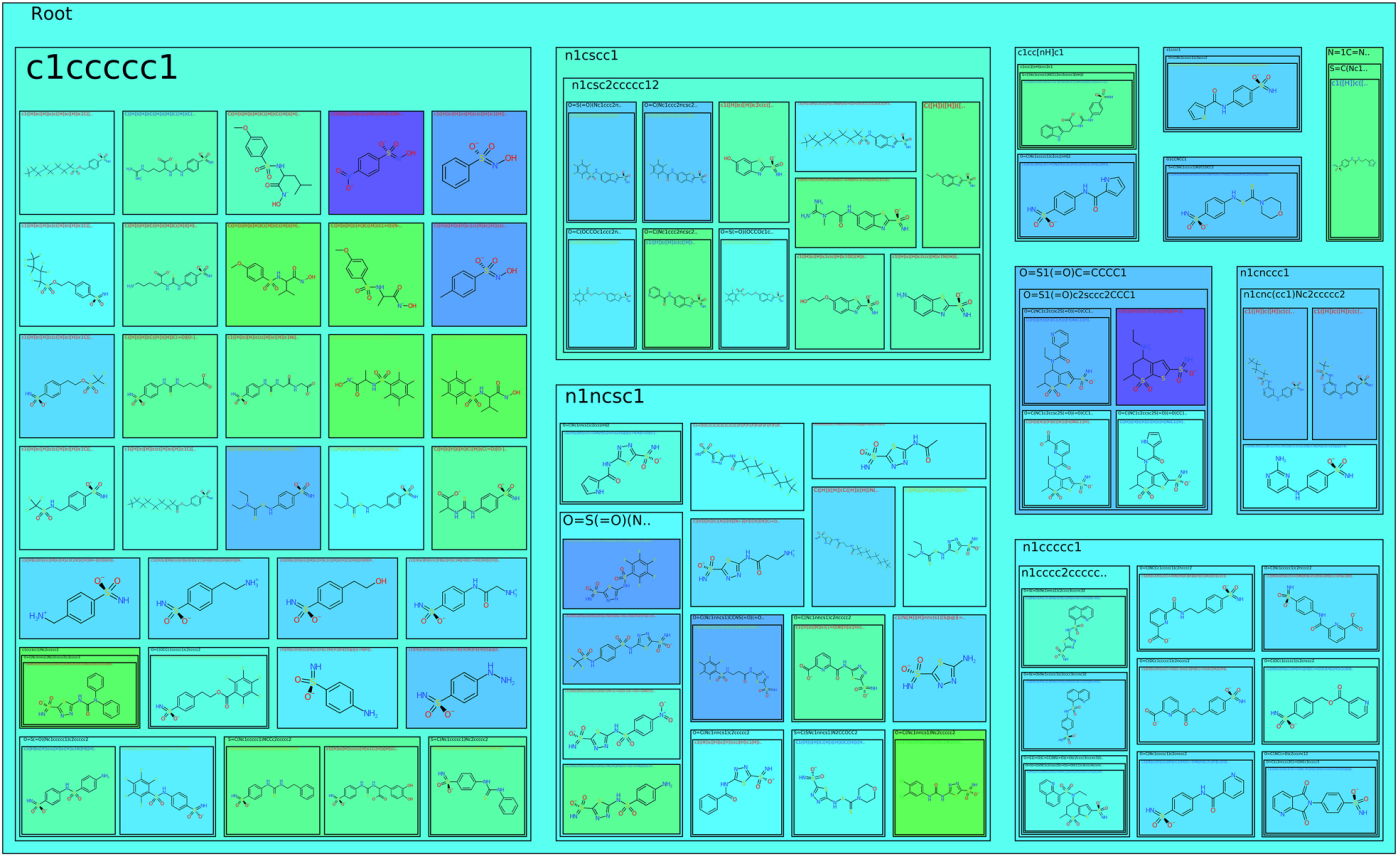
The tree map view with the size of the boxes corresponding to the number of molecules of a scaffold and the *color* according to the $\Delta$pKCAI–CAII. *Green color* indicate less selectivity, whereas *blue color* indicates more selectivity in favor of CAII

**Fig. 12:**
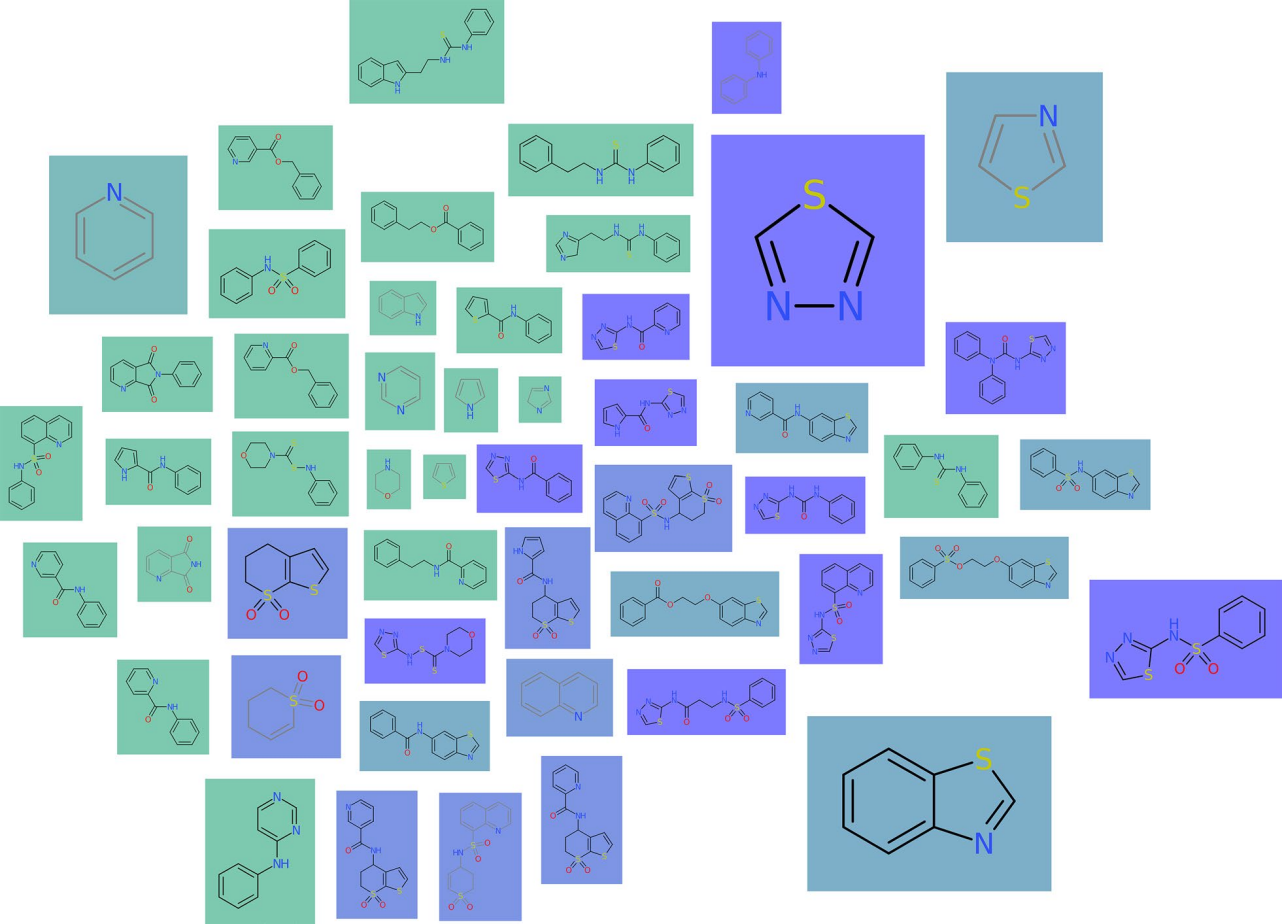
The cloud view maps the similarities of the molecules w.r.t. the three pKi values to a 2D space so as to molecules with similar pKi values are adjacent. The *color* indicates the scaffold id assigned by Weber et al. from *blue* corresponding to scaffold id 1 and *green* corresponding to scaffold id 6

To determine the most selective scaffold, Scaffold Hunter provides the condensed tree map view for the comparison of two isoforms, see Fig. 11. The molecule cloud allows taking all three (or even more) activities into account, see Fig. 12.

## Conclusions and outlook

Scaffold Hunter has evolved from a tool to visualize the scaffold tree to a visual analytics platform for cheminformatics. We presented the most recent developments and new features of the software package. Besides various technical improvements this includes three completely new visualizations as major innovations. The tree map, heat map and molecule cloud view complement the established views and make the software applicable to additional use cases.

Scaffold Hunter supports two approaches to build hierarchical groups of molecules, the scaffold tree and SAHN clustering, which can be configured to use different similarity measures. For future work we plan to develop visualization techniques for the comparison of (hierarchical) clusterings. Moreover, we would like to focus on the scaffold concept and develop more flexible approaches for visualizing their relations. The scaffold tree approach requires to select a single parent scaffold in order to simplify the structural relations. However, for some tasks this is too restrictive and other selection criteria could be useful, e.g., based on bioactivity [43]. Considering all parent relations leads to a directed acyclic graph or a scaffold network [52] and visualization becomes a challenging task. To take this into account, we would like to develop a visualization, which allows the user to adjust the visual complexity in an interactive fashion to find the right balance.

Since workflow environments such as KNIME are enjoying growing popularity in cheminformatics, we plan to integrate individual analysis methods and visualization techniques of Scaffold Hunter into the KNIME platform in the future.

## Availability and requirements

Scaffold Hunter is free software and is available under the GPLv3 License at http://scaffoldhunter.sourceforge.net. The latest version of Scaffold Hunter at the time of publication is 2.6.3, new versions are released on a regular basis. Since Scaffold Hunter is written in Java, it is platform independent and runs on Linux, Mac, Windows and other operating systems. Scaffold Hunter comes as a portable, ready to run bundle, which requires no installation procedure. A special demo mode provides a ready to use small molecular database to try out Scaffold Hunter’s functionality with a single click. In this demo mode, there is no need to import molecular datasets or calculate scaffold trees, fingerprints and other attributes, i.e., to deal with any configuration procedure before using the visual analytic tools. The demo mode uses the dataset and view configurations, which were already used for the case study in section “Case studies using the novel visualization techniques”. Furthermore, the demo mode includes other pre-configured views to show many visual features of Scaffold Hunter and classical use cases. You can download the demo package of the current Scaffold Hunter version here: https://sourceforge.net/projects/scaffoldhunter/files/. If the demo mode convinces you to try out Scaffold Hunter on your own data, it is also easy to set up. For regular use cases, the embedded HSQLDB database backend only requires you the selection a file system location where the imported molecular datasets are stored. Scaffold Hunter is then ready to import molecular datasets (such as SDF-Files), compute scaffold trees and show the above described views on the imported data. Optionally, it is also possible to install a MySQL or MariaDB database server for optimal performance on large datasets and collaborative features.

A simple step by step tutorial to get started with Scaffold Hunter is available at http://scaffoldhunter.sourceforge.net/tutorials.html. If you have any further questions regarding the usage of Scaffold Hunter you can read the bundled manual, have a look at the FAQ http://scaffoldhunter.sourceforge.net/wiki/doku.php?id=faq or ask your question on our user mailing list http://scaffoldhunter-userslists.sourceforge.net.

### Development process

Scaffold Hunter is available as open-source software and participation in the development process is encouraged. The internal organization of the code uses well defined interfaces for all parts of the software. Each public method, class or interface is fully documented using JavaDoc (undocumented code is issued as warning). We use unit testing and have a review process in which each bug-fix or feature update must pass the review of a different developer before being integrated. The software and user documentation is build using Apache Ant.

## References

[1] Wang Y, Suzek T, Zhang J, Wang J, He S, Cheng T, Shoemaker BA, Gindulyte A, Bryant SH (2014). Pubchem bioassay: 2014 update. Nucleic Acids Res.

[2] Humbeck L, Koch O (2017). What can we learn from bioactivity data? Chemoinformatics tools and applications in chemical biology research. ACS Chem Biol.

[3] Wetzel S, Klein K, Renner S, Rauh D, Oprea TI, Mutzel P, Waldmann H (2009). Interactive exploration of chemical space with scaffold hunter. Nat Chem Biol.

[4] Schuffenhauer A, Ertl P, Roggo S, Wetzel S, Koch MA, Waldmann H (2007). The scaffold tree—visualization of the scaffold universe by hierarchical scaffold classification. J Chem Inf Model.

[5] Klein K, Kriege N, Mutzel P (2012) Scaffold Hunter—visual analysis of chemical compound databases. In: Proceedings of the international conference on computer graphics theory and applications and international conference on information visualization theory and applications (GRAPP & IVAPP), pp 626–635

[6] Klein K, Koch O, Kriege N, Mutzel P, Schäfer T (2013). Visual analysis of biological activity data with Scaffold Hunter. Mol Inf.

[7] Ertl P, Rohde B (2012). The molecule cloud—compact visualization of large collections of molecules. J Cheminform.

[8] Spotfire, TIBCO Software Inc. http://spotfire.tibco.com. Accessed 22 March 2017

[9] Berthold MR, Cebron N, Dill F, Gabriel TR, Kötter T, Meinl T, Ohl P, Sieb C, Thiel K, Wiswedel B (2008) In: Preisach C, Burkhardt H, Schmidt-Thieme L, Decker R (eds) KNIME: The Konstanz Information Miner, pp 319–326. Springer, Berlin, Heidelberg (2008). doi:10.1007/978-3-540-78246-9_38

[10] BIOVIA Pipeline Pilot 9.5, Accelrys Software, Inc. http://accelrys.com/products/collaborative-science/biovia-pipeline-pilot/. Accessed 22 March 2017

[11] Wolstencroft K, Haines R, Fellows D, Williams A, Withers D, Owen S, Soiland-Reyes S, Dunlop I, Nenadic A, Fisher P, Bhagat J, Belhajjame K, Bacall F, Hardisty A, de la Hidalga AN, Vargas MPB, Sufi S, Goble C (2013). The Taverna workflow suite: designing and executing workflows of web services on the desktop, web or in the cloud. Nucleic Acids Res.

[12] KNIME cheminformatics extensions. https://tech.knime.org/cheminformatics-extensions. Accessed 22 March 2017

[13] RDKit: Open-source cheminformatics. http://www.rdkit.org. Accessed 22 March 2017

[14] Beisken S, Meinl T, Wiswedel B, de Figueiredo LF, Berthold M, Steinbeck C (2013). Knime-cdk: Workflow-driven cheminformatics. BMC Bioinform.

[15] Indigo Toolkit. http://lifescience.opensource.epam.com/indigo/index.html. Accessed 22 March 2017

[16] Gutlein M, Karwath A, Kramer S (2012). Ches-mapper—chemical space mapping and visualization in 3d. J Cheminform.

[17] Gütlein M, Karwath A, Kramer S (2014). CheS-Mapper 2.0 for visual validation of (Q)SAR models. J Cheminform.

[18] Kuhn T, Willighagen EL, Zielesny A, Steinbeck C (2010). CDK-Taverna: an open workflow environment for cheminformatics. BMC Bioinform.

[19] Truszkowski A, Jayaseelan KV, Neumann S, Willighagen EL, Zielesny A, Steinbeck C (2011). New developments on the cheminformatics open workflow environment CDK-Taverna. J Cheminform.

[20] Warr WA (2012). Scientific workflow systems: pipeline Pilot and KNIME. J Comput Aided Mol Des.

[21] Hilbig M, Rarey M (2015). MONA 2: A light cheminformatics platform for interactive compound library processing. J Chem Inf Model.

[22] Guilloux V, Arrault A, Colliandre L, Bourg S, Vayer P, Morin-Allory L (2012). Mining collections of compounds with screening assistant 2. J Cheminform.

[23] Sander T, Freyss J, Korff Mv, Rufener C (2015). Datawarrior: An open-source program for chemistry aware data visualization and analysis. J Chem Inf Model.

[24] Bertini E, Strobelt H, Braun J, Deussen O, Groth U, Mayer TU, Merhof D (2011) HiTSEE: A visualization tool for hit selection and analysis in high-throughput screening experiments. In: 2011 IEEE symposium on biological data visualization (BioVis), pp 95–102. doi:10.1109/BioVis.2011.6094053 10.1186/1471-2105-13-S8-S4 PMC3355333 22607449

[25] Strobelt H, Bertini E, Braun J, Deussen O, Groth U, Mayer TU, Merhof D (2012). Hitsee knime: a visualization tool for hit selection and analysis in high-throughput screening experiments for the knime platform. BMC Bioinform.

[26] Baell JB, Ferrins L, Falk H, Nikolakopoulos G (2013). PAINS: Relevance to tool compound discovery and fragment-based screening. Aust J Chem.

[27] R Core Team (2016) A language and environment for statistical computing. R Foundation for Statistical Computing, Vienna, Austria (2016). R Foundation for Statistical Computing. Software available at http://www.r-project.org,

[28] Hall M, Frank E, Holmes G, Pfahringer B, Reutemann P, Witten IH (2009). The weka data mining software: an update. ACM SIGKDD Explor Newsl.

[29] Kriege N, Mutzel P, Schäfer T (2014). Practical SAHN clustering for very large data sets and expensive distance metrics. J Graph Algorithms Appl.

[30] Thomas JJ, Cook KA (eds) (2005) Illuminating the path: the research and development agenda for visual analytics

[31] Anderberg MR (1973). Cluster analysis for applications.

[32] Leach AR, Gillet VJ (2007). An introduction to chemoinformatics.

[33] Oellien F, Ihlenfeldt W-D, Gasteiger J (2005). Infvis: platform-independent visual data mining of multidimensional chemical data sets. J Chem Inf Model.

[34] Maniyar DM, Nabney IT, Williams BS, Sewing A (2006). Data visualization during the early stages of drug discovery. J Chem Inf Model.

[35] Borg I (2005). Modern multidimensional scaling: theory and applications.

[36] Barth L, Kobourov SG, Pupyrev S (2014) Experimental comparison of semantic word clouds. In: Gudmundsson J, Katajainen J (eds) Experimental algorithms: 13th international symposium, SEA 2014. Lecture notes in computer science, vol 8504, pp 247–258. Springer, Cham. doi:10.1007/978-3-319-07959-2_21

[37] Johnson B, Shneiderman B (1991) Tree-maps: a space-filling approach to the visualization of hierarchical information structures. In: IEEE conference on visualization, 1991. Visualization ’91, Proceedings, pp 284–291. doi:10.1109/VISUAL.1991.175815

[38] Bajorath J (2014). Exploring activity cliffs from a chemoinformatics perspective. Mol Inform.

[39] Sturm W, Schäfer T, Schreck T, Holzinger A, Ullrich T (2015) Extending the scaffold hunter visualization toolkit with interactive heatmaps. In: Borgo R, Turkay C (eds) Computer graphics and visual computing (CGVC). doi:10.2312/cgvc.20151247

[40] Wilkinson L, Friendly M (2009). The history of the cluster heat map. Am Stat.

[41] Rogers D, Hahn M (2010). Extended-connectivity fingerprints. J Chem Inf Model.

[42] Lachance H, Wetzel S, Kumar K, Waldmann H (2012). Charting, navigating, and populating natural product chemical space for drug discovery. J Med Chem.

[43] Renner S, van Otterlo WAL, Dominguez Seoane M, Möcklinghoff S, Hofmann B, Wetzel S, Schuffenhauer A, Ertl P, Oprea TI, Steinhilber D, Brunsveld L, Rauh D, Waldmann H (2009). Bioactivity-guided mapping and navigation of chemical space. Nat Chem Biol.

[44] Bon RS, Waldmann H (2010). Bioactivity-guided navigation of chemical space. Acc Chem Res.

[45] Dictionary of Natural Products. http://dnp.chemnetbase.com. Accessed 22 March 2017

[46] Oprea TI (2005) (ed):Chemoinformatics in drug discovery. Methods and principles in medicinal chemistry. Wiley-VCH Verlag GmbH & Co. KGaA, Weinheim, FRG. doi:10.1002/3527603743

[47] Wetzel S, Wilk W, Chammaa S, Sperl B, Roth AG, Yektaoglu A, Renner S, Berg T, Arenz C, Giannis A, Oprea TI, Rauh D, Kaiser M, Waldmann H (2010). A scaffold-tree-merging strategy for prospective bioactivity annotation of $$\gamma $$-pyrones. Angew Chem Int Ed Engl.

[48] Muller J, Sperl B, Reindl W, Kiessling A, Berg T (2008). Discovery of chromone-based inhibitors of the transcription factor stat5. Chembiochem Eur J Chem Biol.

[49] Kinase SARfari (2017) https://www.ebi.ac.uk/chembl/sarfari/kinasesarfari. Accessed 22 March

[50] Gaulton A, Bellis LJ, Bento AP, Chambers J, Davies M, Hersey A, Light Y, McGlinchey S, Michalovich D, Al-Lazikani B, Overington JP (2012). ChEMBL: a large-scale bioactivity database for drug discovery. Nucleic Acids Res.

[51] Weber A, Bohm M, Supuran CT, Scozzafava A, Sotriffer CA, Klebe G (2006). 3d qsar selectivity analyses of carbonic anhydrase inhibitors: insights for the design of isozyme selective inhibitors. J Chem Inf Model.

[52] Varin T, Schuffenhauer A, Ertl P, Renner S (2011). Mining for bioactive scaffolds with scaffold networks: improved compound set enrichment from primary screening data. J Chem Inf Model.

[53] Steinbeck C, Hoppe C, Kuhn S, Floris M, Guha R, Willighagen EL (2006) Recent developments of the chemistry development kit (CDK)—an open-source java library for chemo- and bioinformatics. Curr Pharm Des 12(17):2111–2120. Software available at http://cdk.sourceforge.net 10.2174/138161206777585274 16796559

